# Conservation of σ^28^-Dependent Non-Coding RNA Paralogs and Predicted σ^54^-Dependent Targets in Thermophilic *Campylobacter* Species

**DOI:** 10.1371/journal.pone.0141627

**Published:** 2015-10-29

**Authors:** My Thanh Le, Mart van Veldhuizen, Ida Porcelli, Roy J. Bongaerts, Duncan J. H. Gaskin, Bruce M. Pearson, Arnoud H. M. van Vliet

**Affiliations:** Gut Health and Food Safety Programme, Institute of Food Research, Norwich Research Park, Norwich, United Kingdom; Iowa State University, UNITED STATES

## Abstract

Assembly of flagella requires strict hierarchical and temporal control via flagellar sigma and anti-sigma factors, regulatory proteins and the assembly complex itself, but to date non-coding RNAs (ncRNAs) have not been described to regulate genes directly involved in flagellar assembly. In this study we have investigated the possible role of two ncRNA paralogs (CjNC1, CjNC4) in flagellar assembly and gene regulation of the diarrhoeal pathogen *Campylobacter jejuni*. CjNC1 and CjNC4 are 37/44 nt identical and predicted to target the 5' untranslated region (5' UTR) of genes transcribed from the flagellar sigma factor σ^54^. Orthologs of the σ^54^-dependent 5' UTRs and ncRNAs are present in the genomes of other thermophilic *Campylobacter* species, and transcription of CjNC1 and CNC4 is dependent on the flagellar sigma factor σ^28^. Surprisingly, inactivation and overexpression of CjNC1 and CjNC4 did not affect growth, motility or flagella-associated phenotypes such as autoagglutination. However, CjNC1 and CjNC4 were able to mediate sequence-dependent, but Hfq-independent, partial repression of fluorescence of predicted target 5' UTRs in an *Escherichia coli*-based GFP reporter gene system. This hints towards a subtle role for the CjNC1 and CjNC4 ncRNAs in post-transcriptional gene regulation in thermophilic *Campylobacter* species, and suggests that the currently used phenotypic methodologies are insufficiently sensitive to detect such subtle phenotypes. The lack of a role of Hfq in the *E*. *coli* GFP-based system indicates that the CjNC1 and CjNC4 ncRNAs may mediate post-transcriptional gene regulation in ways that do not conform to the paradigms obtained from the Enterobacteriaceae.

## Introduction

The bacterial pathogen *Campylobacter jejuni* is a major cause of foodborne gastroenteritis in the developed world, with infection often associated with the consumption of undercooked poultry products [1]. Clinical symptoms of *C*. *jejuni* infection include watery or bloody diarrhoea, nausea, fever and abdominal pains, although the disease is usually self-limiting in humans [2]. Secondary complications of *C*. *jejuni* infection include the autoimmune diseases Guillain-Barré and Miller-Fisher syndrome that result in paralysis, and *C*. *jejuni* is also associated with reactive arthritis and inflammatory bowel disease [3].

*C*. *jejuni* produces bipolar flagella, which play a central role in virulence and intestinal colonisation. They are required for chemotactic motility, and involved in important processes such as intestinal colonisation, host cell invasion, autoagglutination, and biofilm formation [4–9], and are targeted by bacteriophages [10]. The bacterial flagellum is a multisubunit organelle, which requires the concerted expression and activity of >50 flagellar proteins and accessory factors for correct assembly and function [11, 12], and which in *C*. *jejuni* is also heavily O-glycosylated with pseudaminic and legionaminic acid residues [9, 13, 14]. Flagellar assembly can be roughly distinguished in three separate phases: the early phase, where the flagellar export apparatus and motor-rotor switch complex are assembled in the C and MS rings in the cytoplasmic membrane. This is followed by assembly of the hook- and basal body complex which includes the rod, and then finally the production of the filament from the major flagellin protein FlaA [11, 12]. These processes follow a prescribed hierarchical and temporal order, and interference with any of these steps usually results in a cessation of flagellar assembly.

The three stages of flagellar assembly are controlled at the transcriptional level, via the use of specific promoter sets recognised by separate sigma factors. In *C*. *jejuni*, the early genes are expressed from promoters recognised by the vegetative sigma factor σ^70^, while the middle and late genes are dependent on the alternative sigma factors σ^54^ and σ^28^, respectively [7, 15]. Expression from σ^54^ promoters requires the activation of the FlgRS two-component regulatory system [16, 17], while early expression of σ^28^-dependent genes is inhibited by the FlgM anti-sigma factor [18, 19]. The group of σ^28^-dependent *C*. *jejuni* genes also encode non-flagellar secreted proteins, which have been suggested to play a role in virulence [20–22].

Gene regulation in bacteria is often mediated at the transcriptional level by control of transcription from specific promoters, but is also subject to post-transcriptional control. Non-coding RNAs (ncRNAs) are now well established as major post-transcriptional regulators in bacteria [23], and control mRNA levels and translation, e.g. by blocking access of the ribosome or by targeting the mRNA for degradation by RNaseE, a process which in many bacteria is aided by the Hfq RNA chaperone [23]. While the function of ncRNAs has been well established in model bacteria such as *Escherichia coli* and *Salmonella enterica*, relatively little is known about their role in *C*. *jejuni*.

Recent studies using RNA-sequencing have resulted in the identification of several ncRNA candidates in *C*. *jejuni* [24–30], but with the exception of the CRISPR RNAs [25, 31, 32], a function has not been assigned to any of these ncRNA candidates. In this study, we have selected two *C*. *jejuni* ncRNA candidates named CjNC1 (CJnc10) and CjNC4 (CJnc170) for further study. We show here that CjNC1 and CjNC4 are both expressed from σ^28^ promoters, that they are predicted to target highly conserved sequences in the 5' untranslated region (5' UTR) of σ^54^-dependent transcripts, and are conserved in other thermophilic *Campylobacter* species. Although inactivation of these ncRNAs did not affect flagellar motility or flagella-related phenotypes, reporter gene assays showed that these ncRNAs have the ability to affect translation of specific σ^54^-dependent transcripts in *C*. *jejuni*.

## Materials and Methods

### Bacterial strains, plasmids and growth conditions

*Campylobacter jejuni* NCTC 11168 and isogenic mutants were routinely grown under microaerobic conditions (85% N_2_, 5% O_2_, 10% CO_2_) in a MACS-MG-100 controlled atmosphere cabinet (Don Whitley Scientific), at 37°C. Growth curves were determined by growing *C*. *jejuni* isolates in a FluoStar Omega controlled atmosphere plate reader (BMG Labtech). For this, small volume (200 μl) *C*. *jejuni* cultures were grown in clear, flat-bottomed, 96-well plates under microaerobic conditions at 37°C, shaking at 600 rpm (double orbital), and measurements were taken every 20 min. Broth cultures were carried out in Brucella broth (Becton, Dickinson and Company) with shaking, whereas growth on plates used Brucella agar or Blood Agar Base agar No. 2 with Skirrow supplements (10 μg ml^-1^ vancomycin, 5 μg ml^-1^ trimethoprim, 2.5 IU ml^-1^ polymyxin B). An Innova 4230 incubator (New Brunswick Scientific) was used for shaking aerobic cultures at 37°C. *E*. *coli* strains were cultured in Luria-Bertani (LB) medium at 37°C, with broth cultures shaken at 200 rpm. Where appropriate, media were supplemented with 30 μg ml^-1^ kanamycin, 15 μg ml^-1^ chloramphenicol and/or 100 μg ml^-1^ carbenicillin. All bacterial strains used in this study are given in Table 1, whereas all relevant plasmids are listed in Table 2.

**Table 1:** Bacterial strains used in this study.

Strain	Description ^a^
***C*. *jejuni***	
NCTC 11168	Wild-type *C*. *jejuni* [51]
11168 ΔCjNC1	NCTC 11168 CjNC1::Kan^R^
11168 ΔCjNC4	NCTC 11168 CjNC4::Kan^R^
11168 ΔCjNC1 ΔCjNC4	NCTC 11168 CjNC1::Kan^R^ CjNC4::Cm^R^
11168 CjNC1^ov^	NCTC 11168 *cj0046*::CjNC1^fdxApr^ Cm^R^
11168 CjNC4^ov^	NCTC 11168 *cj0046*::CjNC4^fdxApr^ Cm^R^
11168 Δ*flaAB*	NCTC 11168 (*cj1338-39c*)::Kan^R^ [36]
11168 Δ*fliA*	NCTC 11168 *cj0061*::Kan^R^
***E*. *coli***	
Top10	K-12, general cloning strain
BW25113	K-12, parental strain for Keio collection [50]
JW4130-1	BW25113 *hfq*-722(del)::Kan^R^

a. Kan^R^, kanamycin antibiotic resistance cassette; Cm^R^, chloramphenicol antibiotic resistance cassette; ^fdxApr^, gene under control of the *fdxA* promoter.

**Table 2:** Plasmids used in this study.

Strain	Description ^a^
pXG10	GFP reporter plasmid, low copy number [37]
pZE12-luc	ncRNA expression plasmid, high copy number [37]
pZE-CjNC1	pZE with CjNC1 transcribed from *lac* promoter
pZE-CjNC4	pZE with CjNC4 transcribed from *lac* promoter
pZE-CjNC1mut	pZE with mutated CjNC1 transcribed from *lac* promoter
pZE-CjNC4mut	pZE with mutated CjNC4 transcribed from *lac* promoter
pJV300	pZE with nonsense RNA [37]
pXG10-*cj0428*ATG	pXG10 *cj0428*(5' UTR+ATG)::*gfp*
pXG10-*cj0428*aa1to6	pXG10 *cj0428*(5' UTR+nt 1–15 of gene)::*gfp*
pXG10-*cj0428*aa1to12	pXG10 *cj0428*(5' UTR+nt 1–36 of gene)::*gfp*
pXG10-*cj0428*aa1to20	pXG10 *cj0428*(5' UTR+nt 1–60 of gene)::*gfp*
pXG10-*cj0428*mut	pXG10 *cj0428*(mutated 5' UTR+nt 1–36 of gene)::*gfp*
pXG10-*flgP*	pXG10 *cj1026c*(5' UTR+nt 1–36 of gene)::*gfp*
pXG10-*flaB*	pXG10 *cj1338c*(5' UTR+nt 1–3 of gene)::*gfp*
pXG10-*cj1650*	pXG10 *cj1650*(5' UTR+nt 1–36 of gene)::*gfp*
pXG10-*cj1650*mut	pXG10 *cj1650*(mutated 5' UTR+nt 1–36 of gene)::*gfp*
pXG10-*flgE2*	pXG10 *cj1729c*(5' UTR+nt 1–36 of gene)::*gfp*
pXG10-*flgE2*mut	pXG10 *cj1729c*(mutated 5' UTR+nt 1–36 of gene)::*gfp*
pXG10-*cj0581*	pXG10 *cj0581*(5' UTR+nt 1–36 of gene)::*gfp*

a. *gfp*: gene encoding green fluorescent protein.

### Bioinformatic analyses

Genome sequences were downloaded as FASTA files with contigs or complete genome sequences from the NCBI website (http://www.ncbi.nlm.nih.gov/genome/browse/). Alignments were made with ClustalX2 and MEGA v5.2 [33, 34]. Genomes were searched using BLAST and Artemis. Targets for CjNC1 and CjNC4 were predicted using TargetRNA [35], via the website http://snowwhite.wellesley.edu/targetRNA/. The defaults settings were used, except for the target area which was set to go from -50 to +3 based on the annotated translational start.

### Construction of *C*. *jejuni* CjNC1/CjNC4 inactivation and overexpression mutants

Motile *C*. *jejuni* NCTC 11168 was used to construct genetically modified strains. To make gene deletions and inactivations, kanamycin (Kan^R^) and chloramphenicol (Cm^R^) antibiotic resistance cassettes were used to replace complete genes or to disrupt the gene on the *C*. *jejuni* chromosome. For the CjNC1 and CjNC4 deletions, the flanking regions of the *cj0082*-*cj0085c* and *cj1633*-*cj1634c* genes were amplified with a tag containing a *Bam*HI restriction site, and joined by an overlap PCR and cloned into pGEM-T easy. The resulting plasmids were digested with *Bam*HI and ligated with a Kan^R^ antibiotic resistance cassette. The *C*. *jejuni* ΔCjNC1 ΔCjNC4 double mutant was constructed by inactivating CjNC4 with a Cm^R^ marker in a previously made *C*. *jejuni* ΔCjNC1::Kan^R^ mutant. For overexpression of CjNC1 and CjNC4, the CjNC1 and CjNC4 genes were synthesized with the *fdxA* promoter in a plasmid with *Nco*I restriction sites (GeneArt). The insert was cloned as *Nco*I fragment in plasmid pC46 [36], which contains flanking sequences of the *cj0046* pseudogene and a Cm^R^ marker for selection, which can be used to express *C*. *jejuni* sequences in trans at single copy level. A Δ*fliA* mutant was constructed by replacing the *cj0061c* gene with a Kan^R^ marker. All mutants were confirmed by PCR with primers outside the recombination region, followed by DNA sequencing.

### Construction of *E*. *coli* translational control plasmids

Putative targets for CjNC1 and CjNC4 were tested using the GFP-based reporter system described in [37]. The reporter plasmid pXG10 contains the 5’ UTR of a predicted target fused to a *gfp* gene, and was fused to the 5' UTR of the potential target genes *cj0428*, *flgP*, *flaB*, *cj1650*, *flgE2* and *cj0581*, using complementary oligonucleotides with the required part of the 5’UTRs (S1 Table). For mutated 5' UTRs, oligonucleotides with altered sequences were used. The CjNC1 and CjNC4 ncRNAs expression plasmids were created by digesting plasmid pZE12-luc with *Xba*I, and the 2.2 kb fragment was used as plasmid backbone [37]. CjNC1 and CjNC4 complimentary oligonucleotides were synthesised with a 3’ *Xba*I restriction site, annealed, phosphorylated and ligated in the pZE12 2.2 kb *Xba*I fragment (Table 2). Plasmids were combined in *E*. *coli* Top10 (NEB). Plasmid pJV300, which contains a nonsense ncRNA was used as a negative control [37].

### RNA isolation and Northern hybridisation

RNA was isolated as described previously [24]. RNA samples (10 μg) were denatured in RNA loading buffer (95% (v/v) formamide, 0.1% (w/v) xylene cyanol, 0.1% (w/v) bromophenol blue, 10 mM EDTA) for 5 min at 95°C. They were then separated on 6% acrylamide, 8.3 M urea gels, and transferred to Hybond XL membranes (GE Healthcare) by semi-dry blotter at 25 V for 1 h (Biorad). RNA was UV crosslinked and pre-conditioned in Rapid Hybridisation buffer (GE Healthcare) at 42°C for 1 h, before 5' [γ-32P] end-labeled probes for CjNC1 or CjNC4 were added and incubated for 16 h. Probe sequences are given in S1 Table. Membranes were washed in 5 × SSC/0.1% SDS, 1 × SSC/0.1% SDS and 0.5 × SSC/0.1% SDS. Signals were visualized on a Typhoon 9200 phosphorimager (GE Healthcare) after at least 16 h exposure to a phosphor screen.

### Nested RT-PCR and detection of ncRNAs

Reverse transcription PCR was used to assess σ^28^-dependent transcription of CjNC1 and CjCN4. As these ncRNAs are too short and too similar for the design of a primer pair, we utilised a 5' extension on the reverse primer for cDNA production to introduce an addition primer site. The cDNA was produced with the CjNC1/CjNC4/CjNC3 PCR-tag primers (S1 Table) and Affinity Script (Agilent), followed by a PCR reaction with CjNC1, CjNC3 and CjNC4-specific forward primers and the tag-specific reverse primer. The σ^70^-dependent CjNC3 (CJnc140) ncRNA from the intergenic region between the *cj1258*-*porA* genes [24, 25] was used as a σ^28^-independent, highly transcribed ncRNA control.

### Characterisation of the CjNC1 and CjNC4 deletion and overexpression mutants

Microarray analyses were performed essentially as described previously [38, 39], using custom made Agilent 8×15K arrays with oligonucleotides representing 1608 *C*. *jejuni* genes, as well as the CjNC1 and CjNC4 ncRNAs. Two-dimensional gel electrophoresis was used for proteomic characterisation of the ΔCjNC1 and ΔCjNC4 deletion mutants, using protocols described in [39]. Energy taxis and biofilm assays were performed as described previously [36, 40], using crystal violet as dye.

### Motility and autoagglutination

The A_600_ of an overnight *C*. *jejuni* culture was adjusted to 0.4 using sterile PBS. Bacterial motility was assessed by spotting 10 μl of this culture onto the centre of a 0.4% Brucella agar plate [36]. Plates were photographed after 24, 48, and 72 hours of incubation at microaerobic conditions at 37°C, and the diameter of the halo was measured using ImageJ software (version 1.41; National Institute of Health [http://rsbweb.nih.gov/ij/]). A *C*. *jejuni* NCTC 11168 *flaAB* non-motile mutant was included in all experiments as a negative control [4].

Autoagglutination (cell clumping and sedimentation) was measured by monitoring the A_600_ of a one ml overnight culture in a plastic cuvette, statically incubated at room temperature. All strains were assessed using at least three independent biological replicates. The percentage autoagglutination (% AAG) was calculated as the recorded A_600_ divided by the initial A_600_.

### Invasion assays

The murine intestinal crypt-like cell line m-IC_cl2_ [41] and the colon carcinoma cell line CaCo-2 were cultured as described previously [42]. The cells were grown until confluent on a Type I collagen matrix (C7661, Sigma-Aldrich, UK), in plastic 24-well, flat-bottomed plates (Sarstedt) or on transwell inserts with 8 μm pores (Corning) at 37°C in 5% CO_2_ atmosphere. For transwell inserts the transelectrical resistance of membrane was measured with an epithelial voltohmmeter (EVOM2, World Precision Instruments) and cells were considered confluent when resistance was at least 130 Ω.cm^2^ [41]. For invasion assays, *C*. *jejuni* strains were grown to OD_600_ of 0.3, centrifuged (3,220 *g* for 10 min), resuspended in tissue culture medium and 500 μl was added to the cell monolayers at an MOI of 1,000. Bacterial invasion was allowed for 2 h at 37°C in a 5% CO_2_ atmosphere. Invasion was quantified as described previously [42, 43].

### Microscopy

To visualise flagella, *C*. *jejuni was* grown to OD_600_ of 0.3 in Brucella broth and a 10% dilution was viewed under × 1,000 magnification using an Eclipse 50i microscope (Nikon UK Limited). Flagella were visualised using the Ryu stain [44, 45]. Briefly, two solutions were made: Solution I contained 10 μl 5% phenol solution, 2 g tannic acid, 10 ml saturated aluminium potassium sulphate; Solution II contained 6 g crystal violet in 50 ml ethanol. Solution I and II were mixed in the ratio of 1:10. To the edge of the cover-slip, 5 μl of Ryu stain was applied and left to diffuse into the sample by capillary action. Slides were photographed using a Nikon Coolpix E4500 camera. Scanning electron microscopy was performed essentially as described previously [40].

### Fluorescence measurements

For spectroscopy, *C*. *jejuni* and *E*. *coli* cultures were centrifuged (9,600 *g* for 3 min) and resuspended in PBS to OD_600_ of 0.5. The cell suspension (200 μl) was assayed in triplicate in with a FluoStar OPTIMA plate reader and GFP was excited at 485 nm and detected at 520 nm. For flow cytometry, *E*. *coli* was grown in LB broth for 8 h with shaking, diluted 1:200 with PBS supplemented with 1:1000 diluted propidium iodide solution. Fluorescence was measured in triplicate, in at least three independent experiments by flow cytometry using the Cytomics FC500 MPL (Beckman Coulter) or Eclipse (Sony Biotechnology). Results were analysed using FlowJo (TreeStar) and at least 10,000 live bacteria were included in the analyses.

## Results

### Transcription of the *C*. *jejuni* CjNC1 and CjNC4 non-coding RNAs is σ^28^-dependent

**Fig 1:**
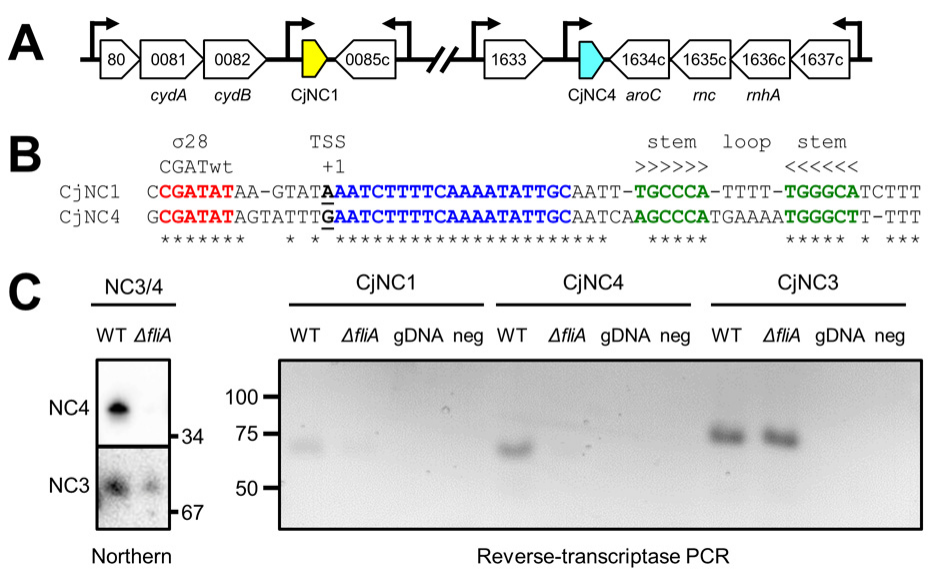
The CjNC1 and CjNC4 non-coding RNAs are paralogs, expressed from σ^28^-dependent promoters. (a) Schematic representation of the genomic position, transcriptional orientation and surrounding genes of the CjNC1 and CjNC4 ncRNAs in *C*. *jejuni* NCTC 11168. Arrows indicate the position of transcription start sites of loci as mapped by dRNA-seq [24]. (b) Alignment and structure prediction of the *C*. *jejuni* NCTC 11168 CjNC1 and CjNC4 ncRNAs. The canonical -10 sequence of the σ^28^-dependent promoter is indicated in red, the transcription start site (TSS) is underlined. The blue residues are the conserved region predicted to interact with target 5' UTRs, and the green residues highlight the complementary nucleotides predicted to form the stem-loop structure which could function as transcriptional terminator. Asterisks indicate conserved nucleotides. (c) Transcription of CjNC1 and CjNC4 is dependent on σ^28^, as shown by comparing transcript levels in wildtype and *fliA* mutant of *C*. *jejuni* NCTC 11168, by Northern hybridisation (left) for CjNC4, and by RT-PCR for CjNC1 and CjNC4 (right), while transcription of the σ^28^-dependent CjNC3 ncRNA is not affected by the inactivation of *fliA*. The RT-PCR utilised a tag-based primer attached by the reverse transcription process (see Methods) and hence cannot amplify genomic DNA, which is includes as control (gDNA). "Neg" represents the negative PCR-control. The addition of the tag adds 29 nt to the size of RT-PCR products.

CjNC1 (CJnc10) and CjNC4 (CJnc170) are two short (<50 nt) ncRNAs which were previously identified by differential RNA-sequencing studies [24, 25]. The CjNC1 ncRNA is found downstream of the *cj0082* (*cydB*) gene [46], whereas the CjNC4 ncRNA is downstream of the *cj1633c* gene (Fig 1A). Comparison of the promoter regions and sequence of CjNC1 and CjNC4 showed that these ncRNAs can be classified as paralogs, as they are 37/44 nt identical. Structure predictions suggested that the second part of both ncRNAs can form a stem-loop structure which could function as Rho-independent transcriptional terminator (Fig 1B). Both ncRNAs are predicted to be transcribed from a σ^28^-dependent promoter, recognised by the CGATwt -10 sequence located upstream of the transcription start site [15, 22, 24, 25]. This was confirmed by comparing transcript levels of CjNC1 and CjNC4 using reverse transcriptase PCR in wildtype *C*. *jejuni* NCTC 11168 and an isogenic *fliA* mutant lacking σ^28^. Transcription of CjNC4 was absent in the *fliA* mutant, whereas there was a very faint band for CjNC1 in the *fliA* mutant. Transcription of a σ^70^-dependent ncRNA (CjNC3, CJnc140) [24, 25] was detected in both the wildtype strain and *fliA* mutant (Fig 1C). CjNC4 was readily detectable in the wildtype strain by Northern hybridisation, but was absent in the *fliA* mutant, while CjNC3 was detected in both wildtype and *fliA* mutant (Fig 1C). We were unable to get reliable detection of CjNC1 using Northern hybridisation.

### CjNC1 and CjNC4 are predicted to target σ^54^-dependent flagellar genes

**Fig 2:**
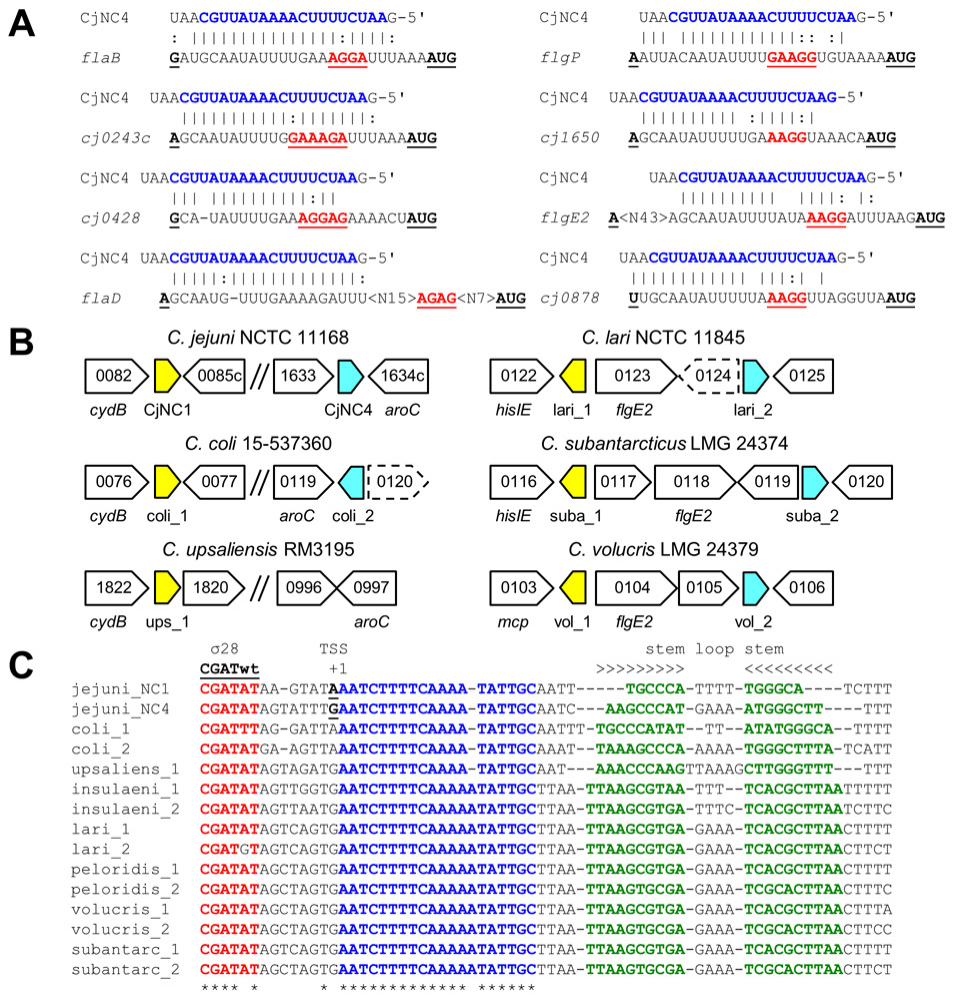
The CjNC1 and CjNC4 ncRNAs are predicted to target the 5' UTRs of σ^54^-dependent genes, and are present in other thermophilic *Campylobacter* species. (a) Alignment of the CjNC4 ncRNA sequence with predicted target 5' UTRs of *C*. *jejuni* σ^54^-dependent genes (*flaB*, *cj0243c*, *c0428*, *flaD*, *flgP*, *cj1650* and *flgE2*) and one σ^70^-dependent gene (*cj0878*), all shown as RNA. The underlined residues indicated the transcription start site (TSS) and AUG startcodon, the predicted ribosome binding site is indicated in red, blue residues the conserved region of the CjNC1 and CjNC4 ncRNAs. Lines indicate complementarity, semicolons highlight U:G pairings, hyphens indicate a gap introduced for optimal alignment. (b) Schematic representation of the *C*. *jejuni* flagellum, modified from [16, 42], with the predicted targets of CjNC1/CjNC4 indicated by a red oval. (c) Comparison of the genomic position and surrounding genes of CjNC1 (yellow) and CjNC4 (blue) orthologs in thermophilic *Campylobacter* species. Dashed lines indicate pseudogenes. Only two examples of the *C*. *lari* group are shown, but all have similar arrangements. (d) Alignment of CjNC1/CjNC4 orthologs of thermophilic *Campylobacter* species. Red residues show the predicted σ^28^ promoter, underlined is the TSS in *C*. *jejuni* NCTC 11168, blue residues show the conserved region predicted to basepair with the 5' UTR targets, and the green residues highlight the complementary nucleotides predicted to form the stem-loop structure which could function as transcriptional terminator. Asterisks indicate conserved nucleotides. Thermophilic *Campylobacter* species included are: jejuni, *C*. *jejuni* NCTC 11168; coli, *C*. *coli* 15–537360, upsaliens, *C*. *upsaliensis* RM3195; insulaeni, *C*. *insulaenigrae* NCTC 12927; lari, *C*. *lari* NCTC 11845; peloridis, *C*. *peloridis* LMG 23910; volucris, *C*. *volucris* LMG 24379; subantarc, *C*. *subantarcticus* LMG 24374.

TargetRNA [35] was used to predict putative targets for the CjNC1 and CjNC4 ncRNAs (Fig 2A, S2 Table), allowing a match with the 5' UTR only. Of the ten highest scoring predictions, seven were previously identified as σ^54^-dependent, and included the genes encoding the minor flagellin FlaB and the hook protein FlgE2 (Fig 2B, S2 Table). Two of the other putative candidates were the *lysC* and *pstB* 5' UTRs, but both genes are within an operon and hence are unlikely to be direct targets, with the other putative target being the σ^70^-dependent *cj0878* gene encoding a small, hypothetical protein. Comparison of the 5' UTRs complementary to the CjNC1 and CjNC4 ncRNAs showed that these were very similar with regard to location and sequence in 7 of the 8 targets, and overlapped with the ribosome binding site, a mode of action which is common among regulatory ncRNAs [23, 37]. The only exception was the *flaD*/*fglL* 5' UTR, which is 69 nt, more than double the length of the average *C*. *jejuni* 5' UTR (30.6 nt) [24], with the predicted interaction site being at the start of the 5' UTR (Fig 2A). We were unable to detect any significant conservation of the predicted interaction area in 5' UTRs of other *C*. *jejuni* σ^54^-dependent genes (not shown).

### CjNC1 and CjNC4 are conserved in thermophilic *Campylobacter* species

To investigate whether this putative ncRNA-regulon is restricted to *C*. *jejuni*, or also present in other *Campylobacter* species, we searched for orthologs of CjNC1 and CjNC4, and the predicted target genes *cj0243c*, *cj0428*, *flaD*, *flgP*, *flaB*, *cj1650* and *flgE2* in the genomes of the thermophilic *Campylobacter* species *C*. *coli*, the *C*. *lari* group (*C*. *lari*, *C*. *insulaenigrae*, *C*. *peloridis*, *C*. *subantarcticus*, *C*. *volucris*) [47], *C*. *upsaliensis*, and the non-thermophilic *Campylobacter* species *C*. *fetus*, *C*. *concisus* and *C*. *curvus*. All the investigated genomes contained orthologs of the σ^54^-dependent target genes, with the exception of *cj0428* (S1 Fig), and included σ^54^ recognition sequences upstream. Comparison of the predicted 5' UTR (based on the position of the σ^54^ consensus sequence) showed that many of the 5' UTRs in the thermophilic *Campylobacter* genomes contain the predicted region of interaction with CjNC1/CjNC4 (S1 Fig, Fig 2A), whereas this region was absent in the non-thermophilic *Campylobacter* species. The *Campylobacter* genome sequences were searched for possible CjNC1/CjNC4 orthologs, based on the presence of a) a σ^28^ -10 sequence, followed by b) the predicted region of interaction, followed by c) a predicted stem-loop structure. Two of each were found in the genomes of the thermophilic *Campylobacter* species, with the exception of *C*. *upsaliensis*, which only contained a single CjNC1/CjNC4 ortholog (Fig 2B), while such sequences were not detected in *C*. *fetus*, *C*. *concisus* or *C*. *curvus*. The location of the ncRNA orthologs differed between the genomes investigated; CjNC1 orthologs of *C*. *jejuni*, *C*. *coli* and *C*. *upsaliensis* were located downstream of the *cj0082* ortholog, while the *C*. *jejuni* and *C*. *coli* CjNC4 orthologs are located downstream of the *aroC* gene (Fig 2B). In contrast, the two CjNC1/CjNC4 orthologs of were both located in the vicinity of the *flgE2* gene of all the members of the *C*. *lari* complex [47]. The predicted ncRNAs showed very strong conservation of the interaction region, with the members of the *C*. *lari* group having an extra A-residue inserted, whereas the downstream stem-loop structure showed more sequence variability (Fig 2C).

### Inactivation or overexpression CjNC1 and/or CjNC4 does not affect flagella-associated phenotypes in *C*. *jejuni*

**Fig 3:**
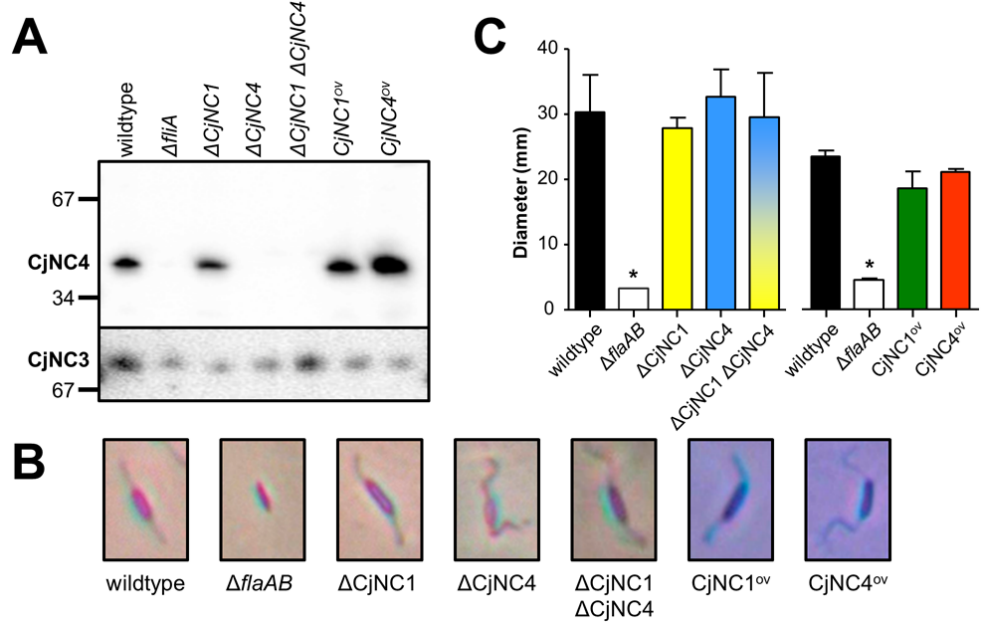
Inactivation and overexpression of CjNC1 and CjNC4 does not affect flagella-related phenotypes in *C*. *jejuni*. (a) The successful inactivation (indicated by Δ) and overexpression (indicated by ^ov^) of CjNC4 is demonstrated using Northern hybridisation. The hybridisation of CjNC3 is shown as loading control. The first two lanes of the Northern hybridisation are also shown in Fig 1C, left panel. (b) Inactivation or overexpression of CjNC1 and CjNC4 does not change cell morphology or production of flagella, as shown by light microscopy (×10,000), with cells and flagella stained by modified Ryu staining [44, 45]. Cutouts show representative cells, the Δ*flaAB* non-motile mutant is included for comparison. (c) Inactivation or overexpression of CjNC1 or CjNC4 does not significantly affect motility on swarm plates, as measured by the diameter of the swarming zone on motility agar plates. Results shown are the average of at least three independent experiments, error bars indicate standard error of the mean. The asterisk indicates *P*<0.05 compared to the wildtype strain (One-way ANOVA). Other phenotypes are reported in S2–S4 Figs.

To investigate the role of the CjNC1 and CjNC4 ncRNAs in *C*. *jejuni*, two sets of mutants were created in *C*. *jejuni* NCTC 11168. One set of mutants were deletion mutants, where CjNC1 or CjNC4 were removed to result in ΔCjNC1 and ΔCjNC4 single mutants, as well as a ΔCjNC1 ΔCjNC4 double mutant. We also cloned the CjNC1 and CjNC4 genes with the *C*. *jejuni fdxA* promoter, which is a strong σ^28^-independent promoter, and inserted this in the *cj0046* pseudogene [36, 48], to achieve overexpression of the individual ncRNAs. Inactivation and overexpression of CjNC4 was confirmed using Northern hybridisation (Fig 3A); we were unable to confirm overexpression of CjNC1.

The morphology of the inactivation mutants and overexpression mutants was characterised by light microscopy, and phenotypes investigated were growth- and flagella-related phenotypes: motility, autoagglutination, energy taxis, biofilm formation and invasion of intestinal epithelial cells. Staining of flagella using modified Ryu-staining [44, 45] showed the presence of flagella in the wildtype strain and the ncRNA inactivation and overexpression mutants, while flagella were absent in a Δ*flaAB* double mutant of *C*. *jejuni* NCTC 11168 (Fig 3B). There was also no effect of ncRNA inactivation or overexpression on motility (Fig 3C), growth at 37°C or 42°C, autoagglutination, energy taxis, biofilm formation (S2 Fig) and invasion of m-IC_cl2_ or Caco-2 intestinal epithelial cells (S3 Fig), consistent with the presence of functional flagella on the mutants. Each experiment included the aflagellated *C*. *jejuni* Δ*flaAB* mutant, which showed the expected phenotypes (increased growth, lack of motility, reduced biofilm formation and lowered invasion of intestinal epithelial cells) [4, 5, 36]. Finally, the effects of CjNC1/CjNC4 inactivation and overexpression on transcript and protein levels was assessed by microarray analysis (S4 Fig, S3 Table) and two-dimensional gel electrophoresis using a subset of the CjNC1 and CjNC4 inactivation and overexpression mutants. Analysis of transcript levels showed a significant reduction of CjNC1 and CjNC4 transcript levels in the ΔCjNC1 ΔCjNC4 double mutant and ΔCjNC4 mutant, but no consistent changes in transcript levels of other *C*. *jejuni* genes. We did not include the ΔCjNC1 single mutant in the microarray analyses, preferring the ΔCjNC1 ΔCjNC4 double mutant which should display any CjNC1-specific changes. The transcript levels of the seven predicted target genes between a 1.6-fold increase and a 1.5-fold decrease, but none of these changes reached statistical significance (S3 Table). Similarly, proteomic characterisation of protein profiles of CjNC1 and CjNC4 inactivation and overexpression mutants did not show any consistent differences with the wildtype strain (data not shown).

### CjNC1 and CjNC4 downregulate translation of target mRNAs in an *E*. *coli*-based GFP reporter system

We hypothesised that regulatory effects of the CjNC1 and CjNC4 ncRNAs could be too subtle to detect with the relatively crude mutagenesis and overexpression methods used in *C*. *jejuni*, and hence decided to isolate the ncRNA and putative targets using an *E*. *coli*-based GFP-reporter system [37]. In this system, the 5' UTR and the first 1 to 20 amino acids of the putative target gene are fused to a *gfp* gene on the low-copy plasmid pXG10, while the candidate ncRNA is cloned behind a *lac* promoter on the compatible high-copy plasmid pZE12, and the two plasmids are co-transformed in *E*. *coli*, followed by measurement of fluorescence in the presence and absence of the ncRNA. A nonsense ncRNA cloned in pZE12 (pJV300) was used as control [37].

**Fig 4:**
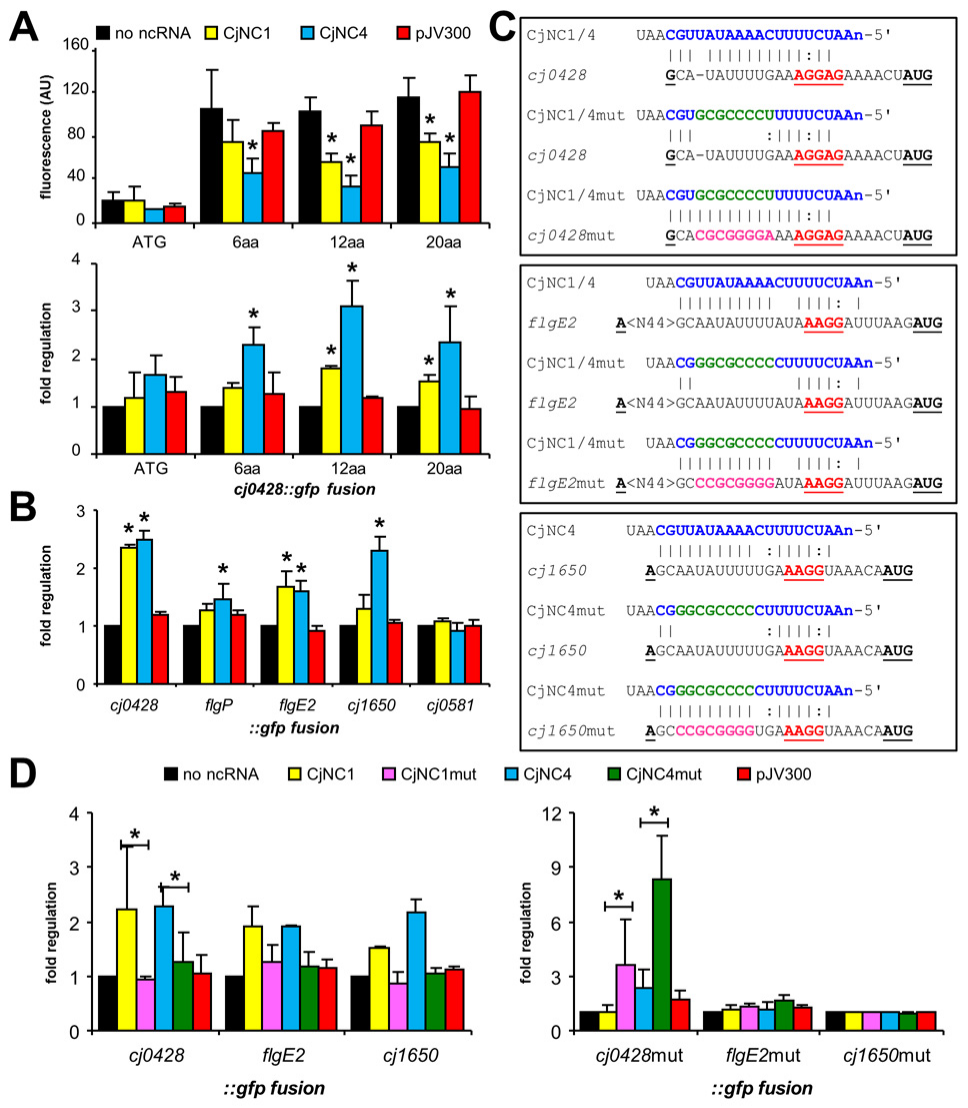
CjNC1 and CjNC4 use sequence-dependent interactions to post-transcriptionally regulate predicted target 5' UTRs in an *E*. *coli* GPF reporter system. (a) Expression of the *cj0428*::*gfp* fusion in pXG10 requires more than just the methionine. The constructs with six or more amino acids of the N-terminus show repression by CjNC1 and CjNC4, while the nonsense ncRNA in pJV300 [37] does not affect fluorescence. Results are shown as flurorescence in arbitrary units (top) and fold regulation (bottom). (b) Expression of *gfp* fusions of *cj0428*, *flgE2*, *cj1650* and *flgP* is repressed by CjNC1 and/or CjNC4, whereas the *cj0581*::*gfp* fusion is not affected by either CjNC1 or CjNC4. None of the fusions is affected by the nonsense ncRNA in pJV300. (c) Mutation strategy of the CjNC1 and CjNC4 ncRNAs, and corresponding regions in the *cj0428*, *flgE2* and *cj1650* 5' UTRs to test whether the observed effects are sequence-specific. The conserved sequence in the ncRNA is shown in blue, the mutations made in the ncRNA in green, the complementary mutations in the 5' UTR in pink. The RBS is indicated in underlined red, the AUG start codon is underlined. The mutated region was chosen to not alter the predicted RBS. (d) Alteration of an 8 nt sequence in CjNC1 and CjNC4 disrupts regulation of the *cj0428*, *flgE2* and *cj1650* 5' UTRs (left), while compensatory mutations in the 5' UTR restore regulation of the *cj0428*::*gfp* fusion (right). The compensatory mutations did not restore regulation of the *flgE2*::*gfp* and *cj1650*::*gfp* fusions, but were associated with a significant reduction in fluorescence (data not shown), and hence we cannot draw any conclusions about the effect of the compensatory mutations on regulation. Data shown are the average of two or more independent experiments, error bars indicate standard error of the mean. Asterisks indicate *P*<0.05 compared to the control with no ncRNA (One-way ANOVA).

The system was first tested using *cj0428*, and fusions were made with different lengths of the Cj0428 N-terminal sequence: only the methionine start, and amino acids 1–6, 1–10 and 1–20. The fusion with methionine start only showed very low fluorescence, while the constructs with amino acids 1–6, 1–10 and 1–20 showed similar levels of fluorescence, which supports the conclusions in [37] which recommended including 10–30 amino acids of the N-terminus in the GFP-fusion (Fig 4A). The *E*. *coli* containing the four *cj0428*::*gfp* fusions were subsequently transformed with the plasmids expressing CjNC1, CjNC4 or the nonsense RNA. There was a significant reduction of fluorescence with especially CjNC4 to ~50%, and with CjNC1 to ~70%, while the nonsense RNA did not affect fluorescence, suggesting that the CjNC1 and CjNC4 can repress translation from the *cj0428* mRNA (Fig 4A).

We subsequently constructed *gfp*-fusions of the putative targets *flaB*, *flgE2*, *flgP*, and *cj1650*, as well as the non-flagellar *cj0581* gene upstream of the *lysC* gene, which was also in the list of possible targets. Although the plasmid was successfully constructed, we were unable to detect any fluorescence from the *flaB*::*gfp* construct (data not shown). The other constructs did fluoresce, with the *cj0581*::*gfp* fusion giving much stronger fluorescence than the other constructs. When transformed with the CjNC1/CjNC4 plasmids, the fluorescence of the *flgE2*::*gfp* fusion was significantly reduced when combined with CjNC1 or CjNC4, and the *cj1650*::*gfp* fusion with CjNC4 (Fig 4B). There was a small reduction of fluorescence with the *flgP*::*gfp* fusion when combined with CjNC4 (Fig 4B). When combined with the nonsense RNA, there was no reduction in fluorescence. The *cj0581*::*gfp* fusion did not show any reduction of fluorescence with the CjNC1, CjNC4 or nonsense RNAs (Fig 4B).

To assess whether the repression of translation observed was sequence-specific, we first mutated the ncRNA sequence of CjNC1 and CjNC4, changing an 8 nt AT-rich sequence downstream of the region complementary to the ribosome binding site in the target mRNA into a GC-rich sequence (Fig 4C). This resulted in an absence of repression by the two mutated ncRNAs (CjNC1mut and CjNC4mut) for the wildtype *cj0428*::*gfp*, *flgE2*::*gfp* and *cj1650*::*gfp* fusions (Fig 4D). The 5' UTRs of these genes were mutated to re-introduce complementarity with the mutated CjNC1mut and CjNC4mut ncRNAs (Fig 4C), and this resulted in significantly reduced fluorescence of the *cj1650mut*::*gfp* and *flgE2mut*::*gfp* fusions. The CjNC1mut and CjNC4mut mutated ncRNAs were able to repress the mutated *cj0428mut*::*gfp*. The wildtype ncRNAs were unable to repress fluorescence of the mutated *gfp* fusions, demonstrating that the repression by CjNC1 and CjNC4 is sequence-specific and dependent on the region of homology predicted (Fig 4D). There was some effect with the *flgE2*::*gfp* fusion, but not with the *cj1650*::*gfp* fusion, and this coincided with reduced fluorescence of these constructs, suggesting that the mutation of the eight residues in the 5' UTR reduces translation of the downstream *gfp* gene, or reduces stability of the mRNA.

### The Hfq RNA chaperone is not required for repression of *cj0428*::*gfp* by CjNC1/CjNC4 in *E*. *coli*

One of the differences between *E*. *coli* and *C*. *jejuni* is the absence of an Hfq RNA chaperone ortholog in Epsilonproteobacteria such as *C*. *jejuni* [49]. To exclude the possibility that the presence of Hfq influences the observed regulation in the *E*. *coli* system, we repeated the experiments with the *cj0428*:*gfp* fusion in wildtype *E*. *coli* K-12 and its isogenic *hfq* mutant obtained from the Keio collection [50]. There was no significant effect of the *hfq* mutation on regulation of the *cj0428*::*gfp* fusion with CjNC1 or CjNC4, although fluorescence was a bit lower in the *hfq* mutant (S5 Fig).

## Discussion

When the first *C*. *jejuni* genome sequence was published in 2000 [51], one of the reported findings was a relative absence of regulatory systems, which suggested at the time that gene regulation in *Campylobacter* species may have lower levels of complexity when compared with other enteric pathogens such as *E*. *coli* and *S*. *enterica*. However, studies using different omics-technologies have since then shown that *C*. *jejuni* has significant regulatory circuitry and is able to modulate expression of its genetic repertoire in response to relevant stimuli [15, 19, 42, 52]. The rapid developments in RNA-sequencing technology allowed the genome-wide mapping of transcriptional start sites and RNA-levels [24–30], while chromatin immunoprecipitation allowed the demonstration of alternative regulatory capacities of well-known regulators such as the Ferric Uptake Regulator [53]. Although *C*. *jejuni* lacks the classical sigma-factors involved in stress-responses (such as RpoS and RpoE), it utilises alternative mechanisms for regulating stress responses, such as multiple transcription start sites and promoters, and the presence of antisense transcription suggests that this may play a role in post-transcriptional regulation of transcript levels in *C*. *jejuni* [24, 25]. Similar observations have been reported for the related human pathogen *Helicobacter pylori*, and ncRNAs have recently been shown to influence gene expression at the post-transcriptional level in *H*. *pylori* [54–56].

Flagellar biogenesis requires a very strict temporal and hierarchical control of the diverse functions. Complex ncRNA networks have been shown to regulate motility in *E*. *coli* [57]. Several ncRNAs have been shown to interact with the 5’UTR of *flhDC* mRNA, which encodes the master regulator of flagellar assembly [58]. These ncRNAs can either be positive or negative regulators of flagellar assembly, and they can undergo positive or negative regulation also in response to the environment. Indirect action through other regulators, such as the ArcB/A two-component system [58] or the CsrA protein post-transcriptional regulator [59], adds further complexity to the network. The Epsilonproteobacteria such as *C*. *jejuni* do not have a FlhDC-like master regulator of flagella, and so there are fundamental differences in the mechanisms that initiate flagellar assembly [60, 61]. *C*. *jejuni* uses its three sigma factors σ^70^, σ^54^ and σ^28^ to control expression of the early, middle and late flagellar genes [15], with σ^54^ requiring the activated NtrC-like regulator FlgR for activity [16, 17], while σ^28^ activity is controlled by the FlgM anti-sigma factor and the correct assembly of the flagellar basal body [18, 19]. The non-coding RNAs CjNC1 and CjNC4 characterised in this study add to the possible complexity of flagellar regulation in *C*. *jejuni*.

Our bioinformatic analyses and experimental data suggest that CjNC1 and CjNC4 are paralogous ncRNAs involved in post-transcriptional regulation of flagellar biogenesis in *C*. *jejuni*. This is based on several independent observations; firstly, the two ncRNAs are transcribed from σ^28^ promoters (Fig 1), which means these will only be transcribed after completion of the basal body, together with the late flagellar genes such as the major flagellin gene *flaA* and several *fed* genes [21, 22]. Secondly, the predicted targets are almost all transcribed from σ^54^-dependent promoters, and include several known flagellar genes such as *flaB*, *flgE2* and *flgP* (Fig 2A), which suggests that CjNC1 and CjNC4 could function as a feedback loop silencing σ^54^-dependent genes. Thirdly, orthologs of CjNC1 and CjNC4 were detected in the other thermophilic *Campylobacter* species *C*. *coli*, the *C*. *lari* group and *C*. *upsaliensis*, and the complementary sequences in the 5' UTRs of the predicted targets are also mostly conserved (Fig 2, S1 Fig). Finally, the ncRNAs were able to mediate partial repression of predicted targets when expressed in an *E*. *coli* GFP reporter system, and that this repression was dependent on the presence of complementary sequences in the ncRNAs and 5' UTRs (Fig 4).

Despite these supportive arguments, it was surprising that we were unable to detect any phenotypes after inactivation and overexpression of the CjNC1 and CjNC4 ncRNAs (Fig 3, S2–S4 Figs). There was no detectable difference in growth and no detectable effect on transcript or protein levels, nor did any of the phenotypic tests known to be flagella-dependent (motility, chemotaxis, biofilm formation, host cell invasion, autoagglutination) show any difference between the deletion and overexpression mutants and the wildtype strain. We would like to offer several possible explanations for this absence of any phenotype. Firstly, *C*. *jejuni* cells primarily need to generate a new flagellum after cell division, and the phenotypic and transcriptomic/proteomic experiments only look at the average of the complete population of cells. As these are not synchronised, this means that possible phenotypes will be masked by the cells not actively expressing the flagellar target genes. Secondly, even in the *E*. *coli* reporter system, the ncRNAs did not completely repress translation from the target genes, and hence the phenotypes may well be below statistical significance. It would require the tracking of transcript and protein levels in a single bacterial cell, and this technology is currently not available for bacteria such as *C*. *jejuni*. Finally, the phenotypic assays used are relatively crude, and more suited for phenotypic differences that are not dependent on temporal changes in the individual bacterial cells, but are consistent within the whole population.

The lack of phenotypic evidence for a role of CjNC1 and CjNC4 makes it difficult to predict the function of this putative regulatory circuit. The most likely function, based on the bioinformatic predictions, is a reduction of translation of σ^54^-dependent genes, equivalent to a feedback-loop, but why the cell would require such a feedback-loop is not clear. Any prediction is further complicated by the lack of information on the role of several of the predicted targets, as no function is currently known for the proteins encoded by *cj0243c*, *cj0428* and *cj1650*. The *cj0428* gene was identified as upregulated during infection of mice [62], and Δ*cj0428* and Δ*cj1650* mutants did not have any motility defects (data not shown). And although FlgP and the coupled protein FlgQ are required for motility [63], their mechanism of action is not known.

The restricted conservation of this regulatory circuit to the thermophilic *Campylobacter* species *C*. *jejuni*, *C*. *coli*, *C*. *lari* group and *C*. *upsaliensis* raises interesting questions on the evolution of this system. *C*. *jejuni*, *C*. *coli* and *C*. *upsaliensis* are more closely related, and hence it was not surprising that the location of the ncRNAs was conserved, with CjNC1 present downstream of the *cydAB* genes [46], and CjNC4 in *C*. *jejuni* and *C*. *coli* downstream and convergently transcribed to the *aroC* gene. The members of the *C*. *lari* group lack orthologs of CydAB, and hence it was not surprising that the CjNC1 and CjNC4 orthologs were elsewhere located, in the vicinity of the *flgE2* gene (Fig 2B). We have previously shown that gene order is poorly conserved in the Epsilonproteobacteria [24], and we therefore do not expect these different genomic locations to have any major effect on their function or expression. We also checked the corresponding genes in several non-thermophilic *Campylobacter* species (*C*. *fetus*, *C*. *curvus and C*. *concisus*) for the possible presence of a CjNC1/CjNC4-like system, and also extended this to *H*. *pylori* and *Helicobacter hepaticus*, but did not find any such system. We could not detect any conserved sequence in the 5' UTR of σ^54^-dependent genes in these genomes, and hence expect this regulatory system to be restricted to the thermophilic *Campylobacter* species.

Although regulation by non-coding RNAs is now well established in many bacteria, it is still poorly understood in the Epsilonproteobacteria, as these lack the RNA chaperone Hfq, which is a central player in regulation by ncRNAs in many bacteria [64–66]. Although the presence of functional analogs of Hfq cannot be excluded, these were not identified in *H*. *pylori* [49], and hence we cannot extrapolate mechanisms of ncRNA-regulation observed in Hfq-positive bacteria to Hfq-negative bacteria such as *C*. *jejuni* and *H*. *pylori*. Two examples of ncRNA-dependent gene regulation have been reported for *H*. *pylori*, as an antisense RNA negatively regulates transcript levels of the urease genes *ureAB* [55], while the C-tract containing small RNA RepG post-transcriptionally regulates the chemotaxis receptor TlpB by interaction with a G-tract in the 5' UTR [54]. Hence we predict that regulation by non-coding RNAs, such as those observed in RNA-sequencing [24–28] will also be shown to play important roles in *C*. *jejuni* biology and virulence. We did show that the interaction of CjNC1 and CjNC4 with the *cj0428* 5' UTR did not require the *E*. *coli* Hfq (S5 Fig), which is consistent with the absence of an Hfq ortholog in *C*. *jejuni*.

## Conclusions

In this study we have shown that thermophilic *Campylobacter* species may have a post-transcriptional control mechanism based on two σ^28^-dependent non-coding RNAs, which are predicted to regulate translation of σ^54^-dependent target genes. This study highlights the complexity of transcriptional regulation in *C*. *jejuni*, which utilises multiple mechanisms to control transcript levels, via multiple promoters and non-coding RNAs. Further investigation is required to dissect the exact role of the reported regulatory function, which may require further developments in single cell-based analysis technologies. Alternatively, studies may focus on the other thermophilic *Campylobacter* species such as the *C*. *lari* group [47] predicted to contain a similar system, to reveal possible roles of ncRNA-dependent flagellar regulation in thermophilic *Campylobacter* species.

## Supporting Information

S1 Fig Alignment of CjNC1/CjNC4 orthologs from thermophilic *Campylobacter* species with the predicted σ^54^-dependent target 5' UTRs. (PDF)

S2 Fig Inactivation and overexpression of CjNC1 and CjNC4 does not affect flagella-related phenotypes in *C*. *jejuni* NCTC 11168. (PDF)

S3 Fig Inactivation and overexpression of CjNC1 and CjNC4 does not affect invasion of intestinal epithelial cells by *C*. *jejuni* NCTC 11168. (PDF)

S4 Fig Inactivation and overexpression of CjNC1 and CjNC4 does not result in consistent changes in transcript levels in *C*. *jejuni* NCTC 11168. (PDF)

S5 Fig Hfq is not required for regulation of the *cj0428*::*gfp* fusion by CjNC1 and CjNC4 in the *E*. *coli* GFP-based reporter system. (PDF)

S1 Table Primers used in this study. (PDF)

S2 Table Predictions by TargetRNA of targets for *C*. *jejuni* CjNC1 and CjNC4. (PDF)

S3 Table Changes in transcript levels of CjNC1 and CjNC4 predicted gene targets in *C*. *jejuni* NCTC11168 CjNC1 and CjNC4 deletion and overexpression mutants. (PDF)
