# Supplementary material for: Conservation of σ28-Dependent Non-Coding RNA Paralogs and Predicted σ54-Dependent Targets in Thermophilic Campylobacter Species
Source: PLoS One. 2015 Oct 29;10(10):e0141627. doi: 10.1371/journal.pone.0141627 (PMC4626219; doi:10.1371/journal.pone.0141627)
Supplement: S1 Fig — (PDF) [file pone.0141627.s001.pdf]

Figure S1

C. coli

flaB

```
 AACGUUAUAAAACUUUUCUAAA-5'
  |||||||||||||||:|||||
GAUGCAAUAUUUUGAAAGGAUUUAAAAUG
```

cj0243

```
    AACGUUAUAAAACUUUUCUAAA-5'
    | |||||||||| :||||||||
AAACUAGCAAUAUUUUUGAAAGAUUUAAAAUG
```

cj0428

```
   AACGUUAUAAAACUUUUCUAAA-5'
   ||||| ||||||||||:||
UUUUUGCA-UAUUUUGAAAGGAGAAAGCUAUG
```

flaD

```
AACGUUAUAAAACUUUUCUAAA-5'
  |||||:||| ||||||||||
 AGCAAUGUUU-GAAAAGAUUU<N18>AGGAUGAAUAUG
```

flgP

```
     AACGUUAUAAAACUUUUCUAAA-5'
     || ||||||||||||:: :|
U<N7>UUACAAUAUUUUGAAGGUGUAAAUAUG
```

cj1650

```
   AACGUUAUAAAA-CUUUUCUAAA-5'
   | ||||||||||  :||||:| |
CUUUAGCAAUAUUUUUUGAAAGGUGUAAAAUG
```

flgE2

```
       AACGUUAUAAAACUUUUCUAAA-5'
         ||||||||||  ||||: ||
U<N41>AAAGCAAUAUUUUAUAAAGGAUUUAAGAUG
```

C. upsaliensis

flaB

```
AACGUUAUAAAACUUUUCUAAG-5'
  ||||||||||||||:||||:
GAGCAAUAUUUUGAAAGGAUUUAAAAUG
```

cj0243

```
AACGUUAUAAAACUUUUCUAAG-5'
  |||||| |||||||||| |
AAGCAAUAAUUUGAAAAGAGUAACAAUG
```

no *cj0428* ortholog

flaD

```
AACGUUAUAAAACUUUUCUAAG-5'
  ||||||||| ||||||||
 AGCAAUAUUU-GAAAAGAUCA<N12>AGGAAAGACAAUG
```

flgP

```
  AACGUUAUAAAACUUUUCUAAG-5'
  || ||||||||||||:: :|
UAUUACAAUAUUUUGAAGGUGUAAAUAUG
```

cj1650

```
AACGUUAUAAAACUUUUCUAAG-5'
  ||||||||| ||||:||  |
 AGCAAUAUUU-GAAAGGAAGCAAAUG
```

flgE2

```
     AACGUUAUAAAA--CUUUU-CUAAG-5'
       |||| |||||  ||||| |: |:
G<N8>AAGCAAGAUUUUAUGAAAAAGGAUUCACUAUG
```

C. lari (*C. lari* group)

flaB

```
 UUCGUUAUAAAAACUUUUCUAAG-5'
 |||||||||| ||||||:||||:
UAAGCAAUAUU-UUGAAAGGAUUUAAAAUG
```

cj0243

```
UUCGUUAUAAAAACUUUUCUAAG-5'
||||||||||||||||:|||
AAGCAAUAUUUUUGAAGAGAAGAGUUAUG
```

no *cj0428* ortholog

flaD

```
 UUCGUUAUAAAAACUUUUCUAAG-5'
 ||  ||| | |||| ::||
UAAAAAAUUUAUUUGCGGAGCAAAAUAAUG
```

flgP

```
UUCGUUAUAAAAACUUUUCUAAG-5'
|| |||     ||  ||||:| :
AAUCAAAUAAAUU--GAAGGUGUAAAAAAUG
```

cj1650

```
UUCGUUAUAAAAACUUUUCUAAG-5'
 |||||||      ::|||:| :
 AGCAAUA--ACAAGGAAGGUGUAUUAUG
```

flgE2

```
      UUCGUUAUAAAAACUUUUCUAAG-5'
      ||||||||||||| |||:||||:
A<N27>AAGCAAUAUUUUUUAAAGGAUUUAAUUAUG
```

C. volucris (*C. lari* group)

flaB
```
 UUCGUUAUAAAAACUUUUCUAAG-5'
 |||||||||| ||||||:||||:
CAAGCAAUAUUAUUGAAAGGAUUUAAAAUG
```

cj0243
```
UUCGUUAUAAAAACUUUUCUAAG-5'
||||||||||||| ||:|||
AAGCAAUAUUUUUAAAGAGAAGAGUUAUG
```

cj0428
```
UUCGUUAUAAAAACUUUUCUAAG-5'
  |:|||| ||||||||||:
 UGUAAUA-UUUUGAAAAGGAGAACACAUG
```

flaD
```
 UUCGUUAUAAAAACUUUUCUAAG-5'
     ||  | |||| ::||
UUUAAAAAUUAUUUGCGGAGCAAGAAAUG
```

flgP
```
UUCGUUAUA--AAAACUUUUCUAAG-5'
|| ||||||   ||  |:: | ||
AAUCAAUAUAAAUUGAAGGUGUUUAAAAUG
```

cj1650
```
UUCGUUAUAAAAACUUUUCUAAG-5'
 |||||||     |:||:|  |
 AGCAAUA-ACAAGGAAGGUGUAUUAUG
```

flgE2
```
       UUCGUUAUAAAAACUUUUCUAAG-5'
       || |||||||||| |||:||||:
A<N25>AAAACAAUAUUUUUUUAAAGGAUUUAAUUAUG
```

C. insulaenigrae (*C. lari* group)

flaB
```
 UUCGUUAUAAAAACUUUUCUAAG-5'
  ||||||||| ||||||:||||:
AUAGCAAUAUUAUUGAAAGGAUUUAAAAUG
```

cj0243
```
UUCGUUAUAAAAACUUUUCUAAG-5'
||||||||||||| ||||||
AAGCAAUAUUUUUAAAAAGAAGAGUCAUG
```

no *cj0428* ortholog

flaD
```
UUCGUUAUAAAAACUUUUCUAAG-5'
   :|| | ||| || :: :||:
   UAAAAAUUUGCACGGAGUUUAAUAUG
```

flgP
```
UUCGUUAUAAAAACUUUUCUAAG-5'
|| |||     |||||:: :||:
AAUCAAAUAAAUUGAAGGUGUUUAAAAUG
```

cj1650
```
UUCGUUAUAAAAACUUUUCUAAG-5'
 |||||||  |   :||:| | :
 AGCAAUA-AUAAAGAAGGUUGUAUUAUG
```

flgE2
```
       UUCGUUAUAAAAACUUUUCUAAG-5'
       || |||||||||| |||:|||||
A<N25>AAAACAAUAUUUUUUUAAAGGAUUCAAUUAUG
```

C. subantarcticus (*C. lari* group)

flaB
```
UUCGUUAUAAAAACUUUUCUAAG-5'
|||||||||||| ||||:||||:
AAGCAAUAUUUU-GAAAGGAUUUAAAAUG
```

cj0243
```
UUCGUUAUAAAAACUUUUCUAAG-5'
||||||||||||||||:|||
AAGCAAUAUUUUUGAAGAGAAGAGUUAUG
```

cj0428
```
UUCGUUAUAAAAA-CUUUUCUAAG-5'
  |:||||||||| ||||:||
  GUAAUAUUUUUUGAAAGGAGAAAACAUG
```

flaD
```
  UUCGUUAUAAAAACUUUUCUAAG-5'
  ||  | ||| | ||:|:  |
AAAAAAAUUAUCUGUGGAGUAAAAUUAUG
```

flgP
```
UUCGUUAUAAAAACUUUUCUAAG-5'
|| |||     |||||:: :|
AAUCAAAUAAAUUGAAGGUGUAAAAAUG
```

cj1650
```
UUCGUUAUAAAAACUUUUCUAAG-5'
 |||||||     |||:: :|
 AGCAAUAACAAGGAAGGUGUAAUCAUG
```

flgE2
```
      UUCGUUAUAAAAACUUUUCUAAG-5'
      ||||||||||||| |||:||||:
A<N28>AAGCAAUAUUUUUUUAAAGGAUUUAAUCAUG
```

Figure S1. Alignment of CjNC1/CjNC4 orthologs from thermophilic *Campylobacter* species with the predicted σ^{54}-dependent target 5' UTRs. Sequences are shown as RNA, lines depict identities, semicolons indicate U:G basepairs. Red residues indicate the predicted ribosome binding site, blue residues the conserved ncRNA part, bold and underlined the predicted AUG startcodon. The +1 of the 5' UTR sequence depicted is based on the distance from the σ^{54} promoter.
