# Supplementary material for: Conservation of σ28-Dependent Non-Coding RNA Paralogs and Predicted σ54-Dependent Targets in Thermophilic Campylobacter Species
Source: PLoS One. 2015 Oct 29;10(10):e0141627. doi: 10.1371/journal.pone.0141627 (PMC4626219; doi:10.1371/journal.pone.0141627)
Supplement: S2 Fig — (PDF) [file pone.0141627.s002.pdf]

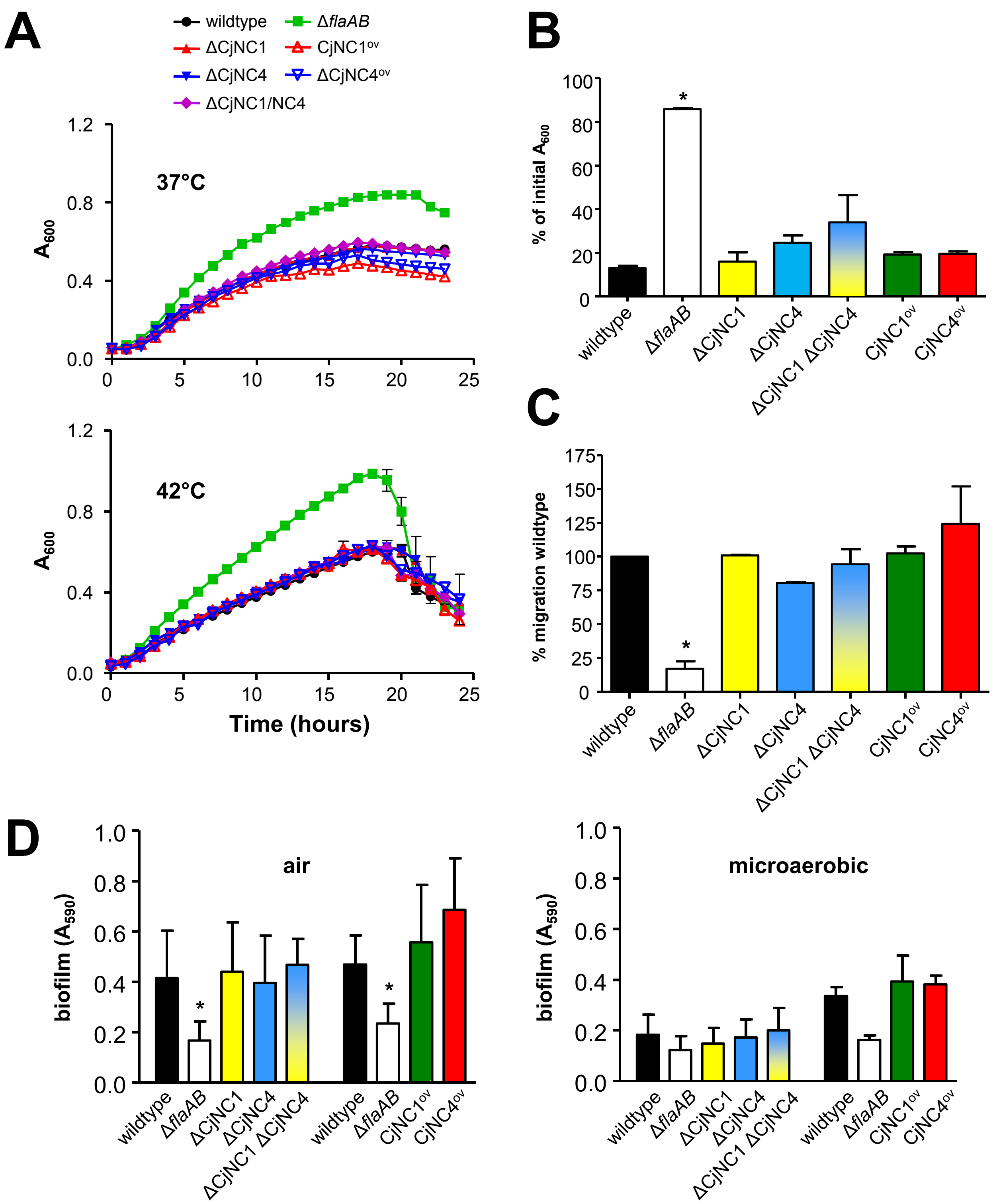

Figure S2. Inactivation and overexpression of CjNC1 and CjNC4 does not affect flagella-related phenotypes in *C. jejuni* NCTC 11168. (A) Growth curves at 37°C and 42°C. (B) Autoagglutination, expressed as the percentage of the initial A_{600} reading. (C) Energy taxis, as measured by the migration into soft agar tubes. (D) Biofilm formation as measured by crystal violet staining of static cultures, incubated for 48h in air (left) and microaerobic conditions (right). Experiments shown are the average of three independent experiments, error bars represent the standard error of the mean. Asterisks indicate $P<0.05$ when compared to the wildtype strain (One-way ANOVA).
