# Supplementary material for: Conservation of σ28-Dependent Non-Coding RNA Paralogs and Predicted σ54-Dependent Targets in Thermophilic Campylobacter Species
Source: PLoS One. 2015 Oct 29;10(10):e0141627. doi: 10.1371/journal.pone.0141627 (PMC4626219; doi:10.1371/journal.pone.0141627)
Supplement: S3 Fig — (PDF) [file pone.0141627.s003.pdf]

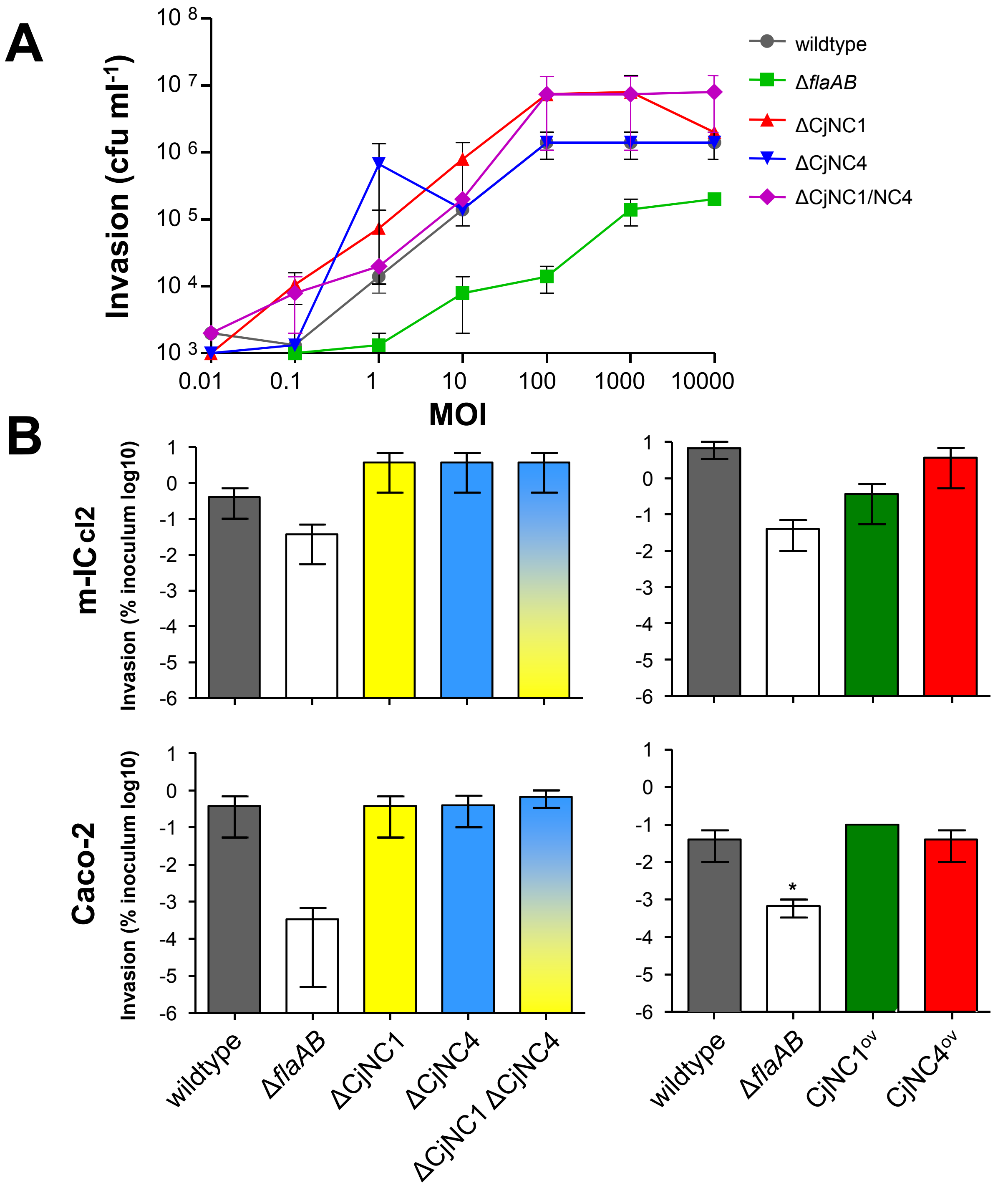

Figure S3. Inactivation and overexpression of CjNC1 and CjNC4 does not affect invasion of intestinal epithelial cells by *C. jejuni* NCTC 11168. Invasion assays were performed, where *C. jejuni* were allowed to invade m-IC$_{cl2}$ for 2 h and Caco-2 monolayers and intracellular bacteria were enumerated by colony counts after a gentamicin wash. A) Invasion of m-ICcl2 monolayers by *C. jejuni* mutants in increasing multiplicities of infection (MOI). Results are expressed in colony forming units per millilitre (cfu ml^{-1}). B) Invasion of m-IC$_{cl2}$ and Caco-2 monolayers by *C. jejuni* strains using an MOI of 1000. Results are expressed as percentage of invaded bacteria with respect to the inocula on a log scale (% inoculum log10). Error bars represent standard error of the mean of data from three biological replicates. Asterisk indicates P < 0.05 relative to WT (One-way ANOVA).
