# Supplementary material for: Conservation of σ28-Dependent Non-Coding RNA Paralogs and Predicted σ54-Dependent Targets in Thermophilic Campylobacter Species
Source: PLoS One. 2015 Oct 29;10(10):e0141627. doi: 10.1371/journal.pone.0141627 (PMC4626219; doi:10.1371/journal.pone.0141627)
Supplement: S4 Fig — (PDF) [file pone.0141627.s004.pdf]

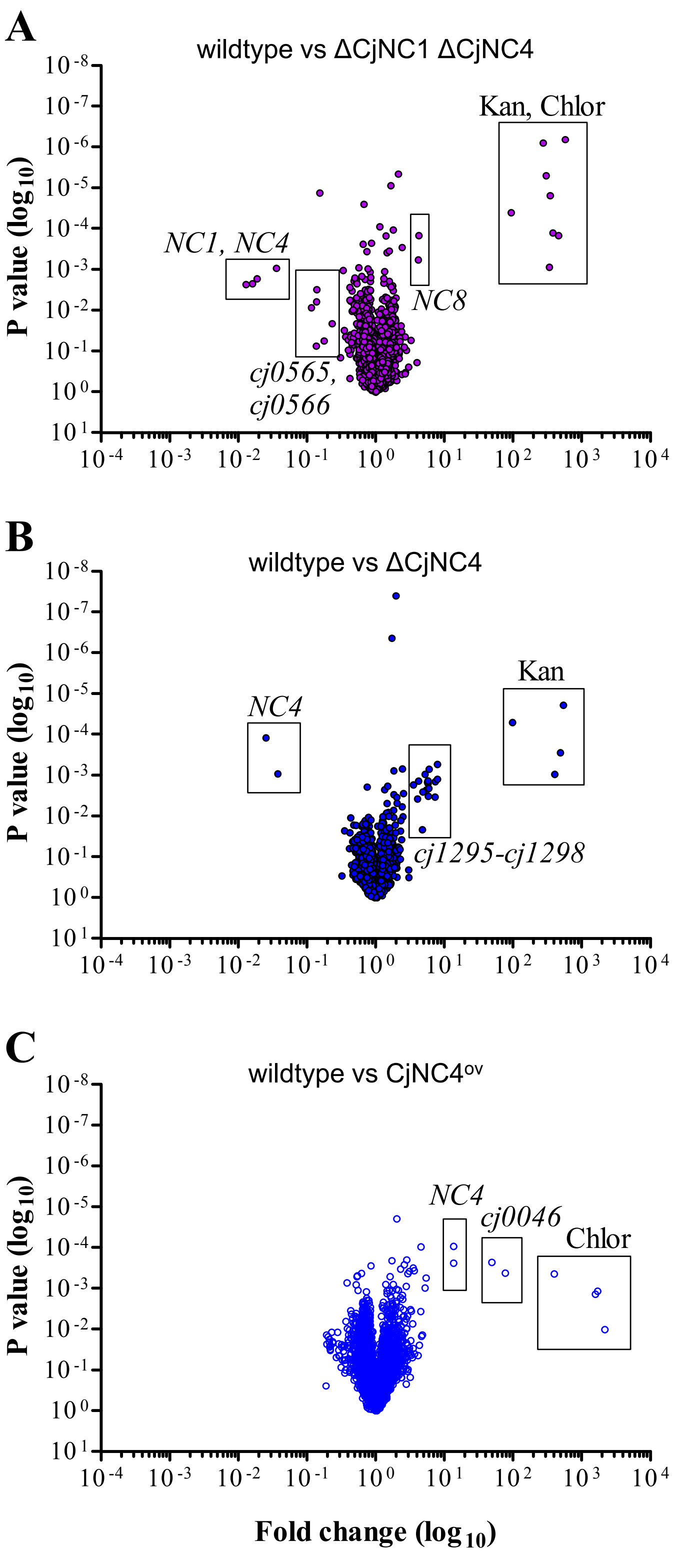

Figure S4. Inactivation and overexpression of CjNC1 and CjNC4 does not result in consistent changes in transcript levels in *C. jejuni* NCTC 11168. Microarrays were used to compare transcript levels the wildtype NCTC 11168 strain to (A) the ΔCjNC1 ΔCjNC4 double mutant, (B) the ΔCjNC4 mutant, and (C) the CjNC4 overexpression strain. The ΔCjNC1 single mutant was not included in this analysis. A summary of changes is represented as volcano plots, where the fold change in gene expression of each gene probe (single point) is plotted against the statistical significance of the change (P value). Each gene has more than one probe and results shown are from two biological replicates. Notable changes in gene expression are labelled.
