# Supplementary material for: Conservation of σ28-Dependent Non-Coding RNA Paralogs and Predicted σ54-Dependent Targets in Thermophilic Campylobacter Species
Source: PLoS One. 2015 Oct 29;10(10):e0141627. doi: 10.1371/journal.pone.0141627 (PMC4626219; doi:10.1371/journal.pone.0141627)
Supplement: S5 Fig — (PDF) [file pone.0141627.s005.pdf]

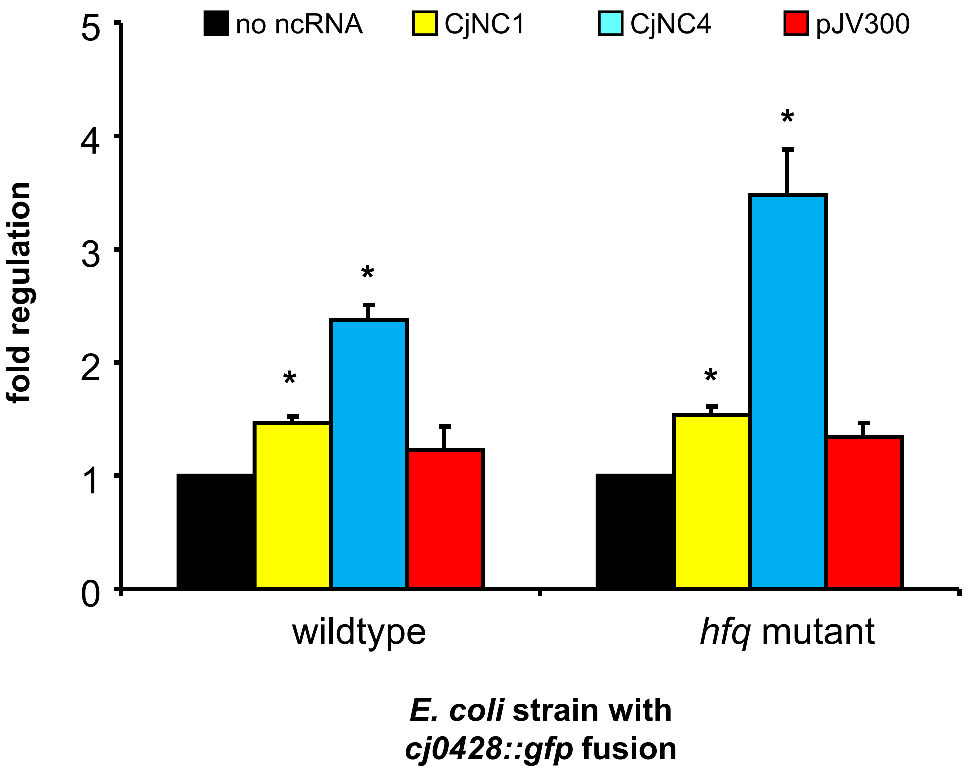

Figure S5. Hfq is not required for regulation of the *cj0428::gfp* fusion by CjNC1 and CjNC4 in the *E. coli* GFP-based reporter system. Wildtype *E. coli* BW25113 (hfq^+) and JW4130-1 (hfq^-) were transformed with the *cj0428::gfp* fusion, and the ncRNA plasmids. Error bars represent standard error of the mean of three independent experiments. Asterisks represent $P < 0.05$ relative to the fluorescence of the fusion without ncRNA (Two-way ANOVA).
