# Supplementary material for: Conservation of σ28-Dependent Non-Coding RNA Paralogs and Predicted σ54-Dependent Targets in Thermophilic Campylobacter Species
Source: PLoS One. 2015 Oct 29;10(10):e0141627. doi: 10.1371/journal.pone.0141627 (PMC4626219; doi:10.1371/journal.pone.0141627)
Supplement: S1 Table — (PDF) [file pone.0141627.s006.pdf]

Table S1. Primers used in this study

Primer name	Sequence (5' - 3') [a]
**RT-PCR detection**	
NC1-Forw-RT	AAATCTTTTCAAAATATTGCAA
NC3-Forw-RT	GAACCGAAAAACATTCATAAG
NC4-Forw-RT	GAATCTTTTCAAAATATTGCAATC
NC1-Rev-tag-RT	GCCTTGCCAGCCCGCTCAGACGAGACATCAAAGATGCCCAAAAATGG
NC3-Rev-tag-RT	GCCTTGCCAGCCCGCTCAGACGAGACATCAGGGATTTAAGCTAGGCGTG
NC4-Rev-tag-RT	GCCTTGCCAGCCCGCTCAGACGAGACATCAAAAAGCCCATTTTCATG
tag-RT	GCCTTGCCAGCCCGCTCAG
**Construction *gfp* plasmids**	
428 UTR F1	GCATATTTTGAAAGGAGAAAACTATGG
428 UTR R1	CTAGCCATAGTTTTCTCCTTTCAAAATATGCTGCA
428 UTR F6	GCATATTTTGAAAGGAGAAAACTATGCAGGTAAATTATAGAG
428 UTR R6	CTAGCTCTATAATTTACCTGCATAGTTTTCTCCTTTCAAAATATGCTGCA
428 UTR F12	GCATATTTTGAAAGGAGAAAACTATGCAGGTAAATTATAGAACGATTAGC TCGTATGAAG
428 UTR R12	CTAGCTTCATACGAGCTAATCGTTCTATAATTTACCTGCATAGTTTTCTCCT TTCAAAATATGCTGCA
428 UTR F20	GCATATTTTGAAAGGAGAAAACTATGCAGGTAAATTATAGAACGATTAGC TCGTATGAATACGATGCTATTAGTGGTCAGTATG
428 UTR R20	CTAGCATACTGACCACTAATAGCATCGTATTCATACGAGCTAATCGTTCTA TAATTTACCTGCATAGTTTTCTCCTTTCAAAATATGCTGCA
LysC UTR F12	CTTGAGATTTAAGGAACAATATTGTGGAAAACGAGAAAAATTATAGACCA AATGTTGCAG
LysC UTR R12	CTGCAACATTTGGTCTATAATTTTTCTCGTTTTCCACAATATTGTTCCTTAA ATCTCAAGTGCA
FlgP UTR F12	AATTACAATATTTTGAAGGTGTAAAAATGAAAAAAATTTATTTTATGCTAG CAATAGCAGGAG
FlgP UTR R12	CTAGCTCCTGCTATTGCTAGCATAAAATAAATTTTTTTCATTTTTACACCTT CAAAATATTGTAATTTGCA
FlaB UTR F1	CGATGCAATATTTTGAAAGGATTTAAAATGG
FlaB UTR R1	CTAGCCATTTTAAATCCTTTCAAAATATTGCATCGTGCA
FlgE2 UTR F12	ATAAACGCAAAAGTTTTTTAAAGCCAAAGCGTTAAATTTTTTAAAGCAATA TTTTATAAAGGATTTAAGATGATGAGATCACTTTGGTCTGGCGTAAGCGGA CTAG
FlgE2 UTR R12	CTAGCTAGTCCGCTTACGCCAGACCAAAGTGATCTCATCATCTTAAATCCT TTATAAAATATTGCTTTAAAAAATTTAACGCTTTGGCTTTAAAAAACTTTT GCGTTTATTGCA
1650 UTR F12	AGCAATATTTTTGAAAGGTAAACAATGAAAAGTGATTTAGATATATTTAA AAAACACTTAG
1650 UTR R12	CTAGCTAAGTGTTTTTTAAATATATCTAAATCACTTTTCATTGTTTACCTTT CAAAAATATTGCTTGCA
NC1 Fwd	AAATCTTTTCAAAATATTGCAATTTGCCCATTTTTGGGCATCTTTT
NC1 Rev	CTAGAAAAGATGCCCAAAAATGGGCAAATTGCAATATTTTGAAAAGATTT
NC4 Fwd	GAATCTTTTCAAAATATTGCAATCAAGCCCATGAAAATGGGCTTTTTT
NC4 Rev	CTAGAAAAAAGCCCATTTTCATGGGCTTGATTGCAATATTTTGAAAAGATT C
428 UTR mut F12	GCACGCGGGGAAAAGGAGAAAACTATGCAGGTAAATTATAGAACGATTA GCTCGTATGAAG

428 UTR mut R12	CTAGCTTCATACGAGCTAATCGTTCTATAATTTACCTGCATAGTTTTCTCCTTT**TCCCCGCG**TGCTGCA
NC1-mut-0428 Fwd	AAATCTTTTC**TCCCCGCG**GCAATTTGCCCATTTTTGGGCATCTTTT
NC1-mut-0428 Rev	CTAGAAAAGATGCCCAAAAATGGGCAAATTGC**CGCGGGGA**GAAAAGATTT
NC1-mut-0428 Fwd	GAATCTTTTC**TCCCCGCG**GCAATCAAGCCCATGAAAATGGGCTTTTTT
NC1-mut-0428 Rev	CTAGAAAAAGCCCATTTTCATGGGCTTGATTGC**CGCGGGGA**GAAAAGATTC
FlgE2 UTR mut F12	ATAAACGC**CCGCGGGG**TTTAAAGCCAAAGCGTTAAATTTTTTAAAGCAATATTTTATAAAGGATTTAAGATGATGAGATCACTTTGGTCTGGCGTAAGCGGACTAG
FlgE2 UTR mut R12	CTAGCTAGTCCGCTTACGCCAGACCAAAGTGATCTCATCATCTTAAATCCTTTATAAAATATTGCTTTAAAAAATTTAACGCTTTGGCTTTAAA**CCCCGCGG**GCGTTTATTGCA
NC1-mut-flgE2 Fwd	AAATCTTTTC**CCCCGCGG**GCAATTTGCCCATTTTTGGGCATCTTTT
NC1-mut-flgE2 Rev	CTAGAAAAGATGCCCAAAAATGGGCAAATTGC**CCGCGGGG**GAAAAGATTT
NC1-mut-flgE2 Fwd	GAATCTTTTC**CCCCGCGG**GCAATCAAGCCCATGAAAATGGGCTTTTTT
NC1-mut-flgE2 Rev	CTAGAAAAAGCCCATTTTCATGGGCTTGATTGC**CCGCGGGG**GAAAAGATTC

a. Red residues indicate the 5' UTR of the genes, blue residues are 5' and 3' added nucleotides for cloning or RT-PCR purposes, green underlined residues are altered residues.
