# Supplementary material for: Conservation of σ28-Dependent Non-Coding RNA Paralogs and Predicted σ54-Dependent Targets in Thermophilic Campylobacter Species
Source: PLoS One. 2015 Oct 29;10(10):e0141627. doi: 10.1371/journal.pone.0141627 (PMC4626219; doi:10.1371/journal.pone.0141627)
Supplement: S2 Table — (PDF) [file pone.0141627.s007.pdf]

**Table S2. Predictions by TargetRNA [a] of targets for *C. jejuni* CjNC1 and CjNC4**

| CjNC1 | | | CjNC4 | | |
|---|---|---|---|---|---|
| **Rank** | **Gene** | **P value** | **Rank** | **Gene** | **P value** |
| 1 | *cj1338c* (*flaB*)* | 3.21 x$10^{-7}$ | 1 | *cj1338c* (*flaB*)* | 9.28 x$10^{-7}$ |
| 2 | *cj0243c** | 9.28 x$10^{-7}$ | 2 | *cj0243c** | 2.68 x$10^{-7}$ |
| 3 | *cj1026c* (*flgP*)* | 6.51 x$10^{-5}$ | 3 | *cj0878* | 8.04 x$10^{-5}$ |
| 4 | *cj0878* | 8.05 x$10^{-5}$ | 4 | *cj0428** | 9.95 x$10^{-5}$ |
| 5 | *cj0428** | 9.95 x$10^{-5}$ | 5 | *cj0582* (*lysC*) | 1.88 x$10^{-4}$ |
| 6 | *cj1729c* (*flgE2*)* | 1.23 x$10^{-4}$ | 6 | *cj1026c* (*flgP*)* | 1.88 x$10^{-4}$ |
| 7 | *cj0582* (*lysC*) | 1.88 x$10^{-4}$ | 7 | *cj0852c* | 2.33 x$10^{-4}$ |
| 8 | *cj0143c* | 2.33 x$10^{-4}$ | 8 | *cj1650** | 2.88 x$10^{-4}$ |
| 9 | *cj0852c* | 2.33 x$10^{-4}$ | 9 | *cj1729c* (*flgE2*)* | 3.56 x$10^{-4}$ |
| 10 | *cj1650** | 2.88 x$10^{-4}$ | 10 | *cj0143c* | 4.41 x$10^{-4}$ |
| 11 | *cj0462* | 5.45 x$10^{-4}$ | 11 | *cj0462* | 5.45 x$10^{-4}$ |

a. Predictions done using default settings, except for an extension of the 5' search area to 50 nt upstream of the ATG startcodon, using the TargetRNA webiste [1] (http://snowwhite.wellesley.edu/targetRNA/).

1. Tjaden B. TargetRNA: a tool for predicting targets of small RNA action in bacteria. Nucl Acids Res. 2008;36(Web Server issue):W109-13. doi: 10.1093/nar/gkn264. PubMed PMID: 18477632.
