# Supplementary material for: Conservation of σ28-Dependent Non-Coding RNA Paralogs and Predicted σ54-Dependent Targets in Thermophilic Campylobacter Species
Source: PLoS One. 2015 Oct 29;10(10):e0141627. doi: 10.1371/journal.pone.0141627 (PMC4626219; doi:10.1371/journal.pone.0141627)
Supplement: S3 Table — (PDF) [file pone.0141627.s008.pdf]

Table S3. Changes in transcript levels of CjNC1 and CjNC4 predicted gene targets in *C. jejuni NCTC11168* CjNC1 and CjNC4 deletion and overexpression mutants.

	**ΔCjNC4**		**ΔCjNC1 ΔCjNC4**		**$CjNC4^{ov}$**	
Gene	Fold change	P value	Fold change	P value	Fold change	P value
cj0243c	-1.45	0.72	-1.32	0.20	1.07	0.73
cj0428	-1.09	0.51	-1.33	0.07	1.63	0.06
cj0887c (*flaD*/*flgL*)	-1.26	0.37	1.08	0.59	-1.08	0.24
cj1026c (*flgP*)	-1.08	0.44	1.05	0.45	1.24	0.11
cj1338c (*flaB*)	-1.48	0.30	-1.37	0.50	-1.06	0.14
cj1650	1.01	0.45	-1.09	0.50	1.14	0.36
cj1729c (*flgE2*)	-1.05	0.60	1.04	0.70	1.26	0.26
